# Selective targeting of nuclear receptor FXR by avermectin analogues with therapeutic effects on nonalcoholic fatty liver disease

**DOI:** 10.1038/srep17288

**Published:** 2015-12-01

**Authors:** Lihua Jin, Rui Wang, Yanlin Zhu, Weili Zheng, Yaping Han, Fusheng Guo, Frank Bin Ye, Yong Li

**Affiliations:** 1 State Key Laboratory of Cellular Stress Biology, Innovation Center for Cell Signaling Network, School of Life Sciences, Xiamen University, Fujian 361005, China

## Abstract

Non-alcoholic fatty liver disease (NAFLD) has become a predictive factor of death from many diseases. Farnesoid X receptor (FXR) is an ideal target for NAFLD drug development due to its crucial roles in lipid metabolism. The aim of this work is to examine the molecular mechanisms and functional roles of FXR modulation by avermectin analogues in regulating metabolic syndromes like NAFLD. We found that among avermectin analogues studied, the analogues that can bind and activate FXR are effective in regulating metabolic parameters tested, including reducing hepatic lipid accumulation, lowering serum cholesterol and glucose levels, and improving insulin sensitivity, in a FXR dependent manner. Mechanistically, the avermectin analogues that interact with FXR exhibited features as partial agonists, with distinctive properties in modulating coregulator recruitment. Structural features critical for avermectin analogues to selectively bind to FXR were also revealed. This study indicated that in addition to antiparasitic activity, avermectin analogues are promising drug candidates to treat metabolism syndrome including NAFLD by directly targeting FXR. Additionally, the structural features that discriminate the selective binding of FXR by avermectin analogues may provide a unique safe approach to design drugs targeting FXR signaling.

Non-alcoholic fatty liver disease (NAFLD), caused by accumulation of abnormal amounts of fat in the liver not due to excessive alcohol use, has emerged as a serious metabolic disorder. Patients with NAFLD have a variety of hepatic dysregulation ranging from abnormal triglyceride accumulation in hepatocytes (steatosis) to steatohepatitis (non-alcoholic steatohepatitis, NASH) with fibrosis, which may evolve to cirrhosis and hepatocellular carcinoma^1^,^2^. Hepatic steatosis is present in up to one-third of adults in developed countries including increasing young people, directly contributing to many liver diseases^2^,^3^. For example, NAFLD-induced liver failure is a leading indication for liver transplantation^4^. Moreover, NAFLD has become a meaningful predictive factor of death from cardiovascular diseases, as well as of the onset of type 2 diabetes and chronic kidney diseases^5^,^6^. These considerations strongly indicate the necessity to treat each stage of NAFLD. Unfortunately, limited pharmacological intervention specific for NAFLD is available for the effective control and treatment of this metabolic disorder.

The nuclear bile acid receptor, farnesoid X receptor (FXR), plays critical roles in maintaining the homeostasis of bile acids, lipid and glucose, as well as in the regulation of inflammation and cancer^7^,^8^,^9^. FXR is highly expressed in liver, intestine, kidney and adrenal gland^7^. Interestingly, there are increasing evidences indicating the importance of FXR function in NAFLD^10^,^11^,^12^,^13^. The expression of FXR was markedly reduced in livers of obese rodents and NAFLD patients^11^,^12^,^13^. In addition, the knockout of FXR in mice caused hepatic steatosis and hyperlipidemia, which could be efficiently improved by overexpression or activation of FXR^14^,^15^,^16^,^17^. Evidence that hepatic expression of aldo-keto reductase 1b7 (*AKR1B7*), a direct target gene of FXR, significantly lowers hepatic triglyceride and cholesterol levels in db/db mice further emphasized the importance of FXR in regulating lipid metabolism^18^. Treatment with FXR ligands, either by intraperitoneal (i.p.) injection or oral administration, can reduce the triglyceride level in liver tissue, resulting in the alleviation of fat accumulation in liver^15^,^16^,^19^,^20^, suggesting the therapeutic effects of FXR agonists in NAFLD.

A natural FXR ligand, chenodeoxycholic acid (CDCA), has been used to treat cholesterol gallstones in human, and must be administrated at least for half a year. However, a CDCA-rich diet has been reported to induce liver hypertrophy in mice^21^. In addition, the binding affinity of CDCA is very low, and it does not target FXR specifically in mammalians^7^. Administration of a synthetic ligand, GW4064, resulted in weight gain and fat accumulation in mice^22^, and even hepatobiliary injury to medaka eleutheroembryo^23^. In addition, INT-747, PX-102 and WAY-362450 are synthetic FXR agonists that are currently under development for treating dyslipidemia, diabetes, primary biliary cirrhosis or NASH, but safety issue remains a major concern for these synthetic molecules.

The avermectins are a series of 16-membered macrocyclic lactone derivatives generated as fermentation products by a soil actinomycete *Streptomyces avermitilis*^24^. Since their potent anthelmintic and insecticidal properties, a series of derivatives were synthesized from avermectin, including ivermectin (Ivomec®), doramectin (Dectomax®), abamectin (Virbamec®) and eprinomectin (Ivomec® Eprinex®), which are all widely used as antiparasitic drugs in animals and/or humans^25^,^26^. Additionally, there were other two kinds of macrocyclic lactones, moxidectin (Cydectin®), which is a semisynthetic derivative of macrocyclic lactone milbemycin (Interceptor® or Sentinel®) produced by soil actinomycete *Streptomyces cyanogriseus*^27^. Moxidectin and milbemycin were used to kill parasites as alternatives to avermectins^27^.In our previous study, we found that ivermectin decreased the serum glucose and cholesterol levels in diabetic mice, by binding and activating nuclear receptor FXR with high affinity and selectivity^28^.

In present study, we further explored the therapeutic effects of ivermectin in treating metabolic disorders and underlying mechanism. Consistent with its effects in regulating glucose and cholesterol, ivermectin treatment effectively reduced fat accumulation in diabetic mice. We further assessed whether other avermectin analogues, also as antiparasitic drugs, have similar effects as ivermectin. Interestingly, the potency of avermectin analogues in regulating metabolism correlates with their FXR binding capabilities, and only the analogues that can bind and activate FXR reduced fat accumulation in mice liver. Mechanistic studies further revealed that avermectin analogues interacting with FXR exhibited features as partial agonists, which allows the differential recruitment of both coactivators and corepressors. Our results suggest that avermectin analogues possess potent therapeutic effects on NAFLD through FXR signaling.

## Results

### Avermectin analogues have disparate capabilities in reducing lipid accumulation in mice

**Figure 1:**
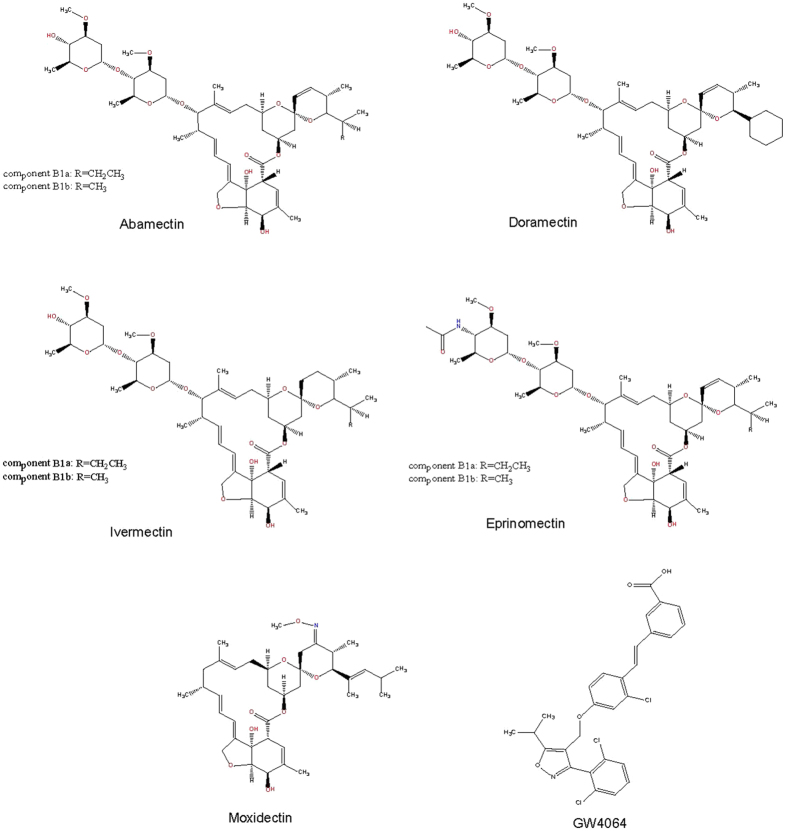
Chemical structures of compounds used in the study.

**Figure 2:**
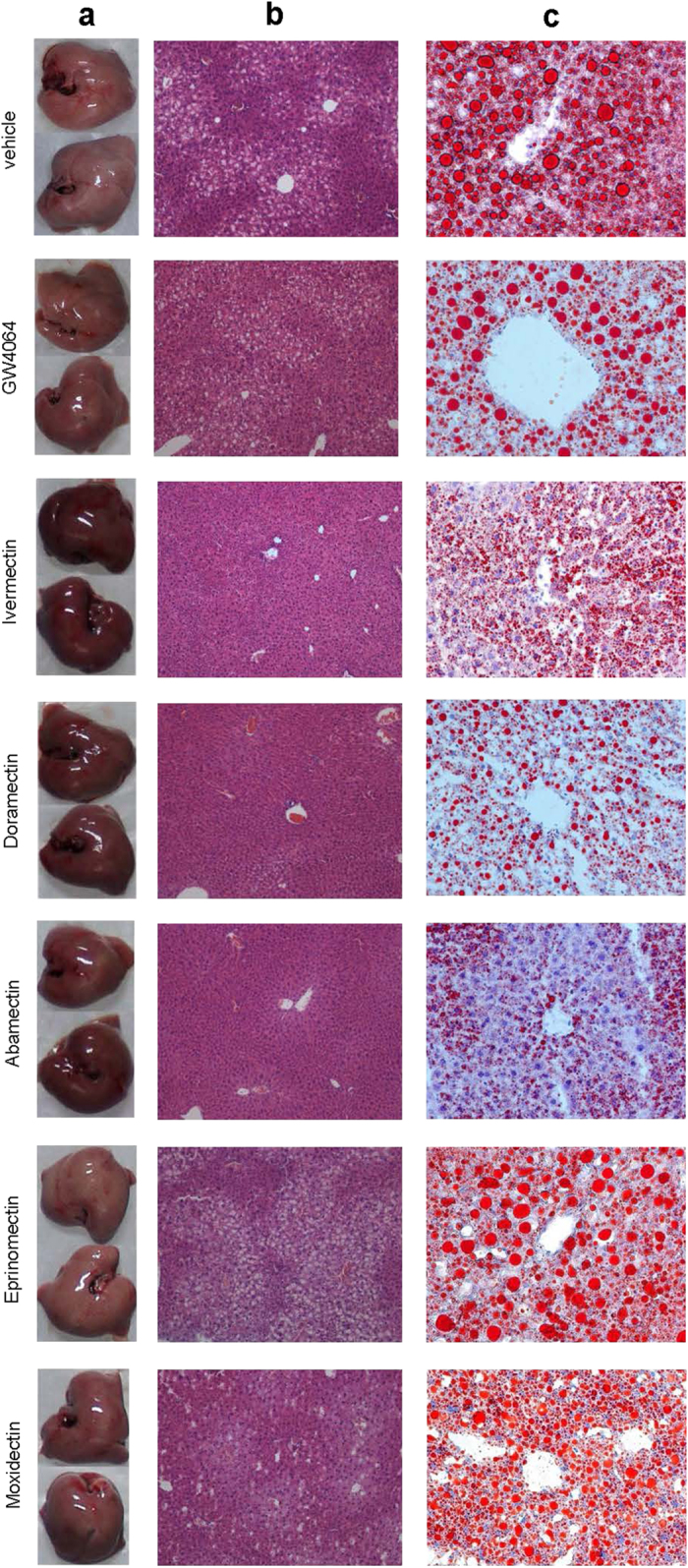
Effects of avermectin analogues on liver fat. (**a**) The liver morphology of mice. (**b**) H&E staining of liver sections (original magnification, ×200). (**c**) Oil Red O staining of liver sections (original magnification, ×200).

In our previous study, we found that the antiparasitic drug ivermectin can target mammalian FXR to regulate cholesterol and glucose metabolism, which make us wonder whether this unique antiparasitic FXR ligand has therapeutic effects on NAFLD by regulating lipid metabolism, and whether other macrocyclic lactone analogues have similar effects on metabolism. Ivermectin together with abamectin, doramectin, eprinomectin and moxidectin selected from avermectins and milbemycins, respectively (Fig. 1), were used to investigate the physiological effects in diabetic mice. 10-week age male KK-Ay mice were fed with high-fat diet and intraperitoneally (i.p.) injected once daily with vehicle, GW4064 and compounds mentioned above. After compound treatment for 14 days, the liver from mice treated with various compounds displayed obvious differences in both morphology and pathology. Remarkably, the livers of mice treated with ivermectin, doramectin and abamectin showed much more fresh red tissue (Fig. 2a). In contrast, the liver color of mice treated with vehicle, eprinomectin and moxidectin was grease white, whereas the livers of mice treated with GW4064 showed a little fresh red. We then performed haematoxylin and eosin (H&E) staining of the paraffin-embedded liver sections, which revealed similar results and explained the observations above (Fig. 2b). Histological examination of liver sections obtained from vehicle treated KK-Ay mice showed the extensive existence of vesicular hepatocyte vacuolation, while GW4064 slightly reduced the hepatic lipid. However, ivermectin, doramectin and abamectin treatment nearly completely reverse the liver from hepatic steatosis in the diabetic mice with the disappeared hepatic lipid accumulation and tight compact structure of the liver cells. In contrast, hepatocellular vacuolation was similar or worse in liver treated with moxidectin and eprinomectin compared with vehicle control. To further confirm the lipid accumulation, we performed oil red O staining of the frozen liver sections (Fig. 2c). As expected, liver sections from vehicle treated mice showed abundant lipid accumulation, especially containing many large lipid droplets. GW4064 treatment slightly reduced the lipid accumulation, while liver sections from mice treated with ivermectin, abamectin and doramectin dramatically reduced the lipid accumulation, where the large lipid droplets nearly disappeared. In contrast, the lipid accumulation was similar with the vehicle control in liver sections of mice treated with moxidectin and eprinomectin.

### Effects of avermectin analogues on various metabolic parameters correlate with liver histology

**Figure 3:**
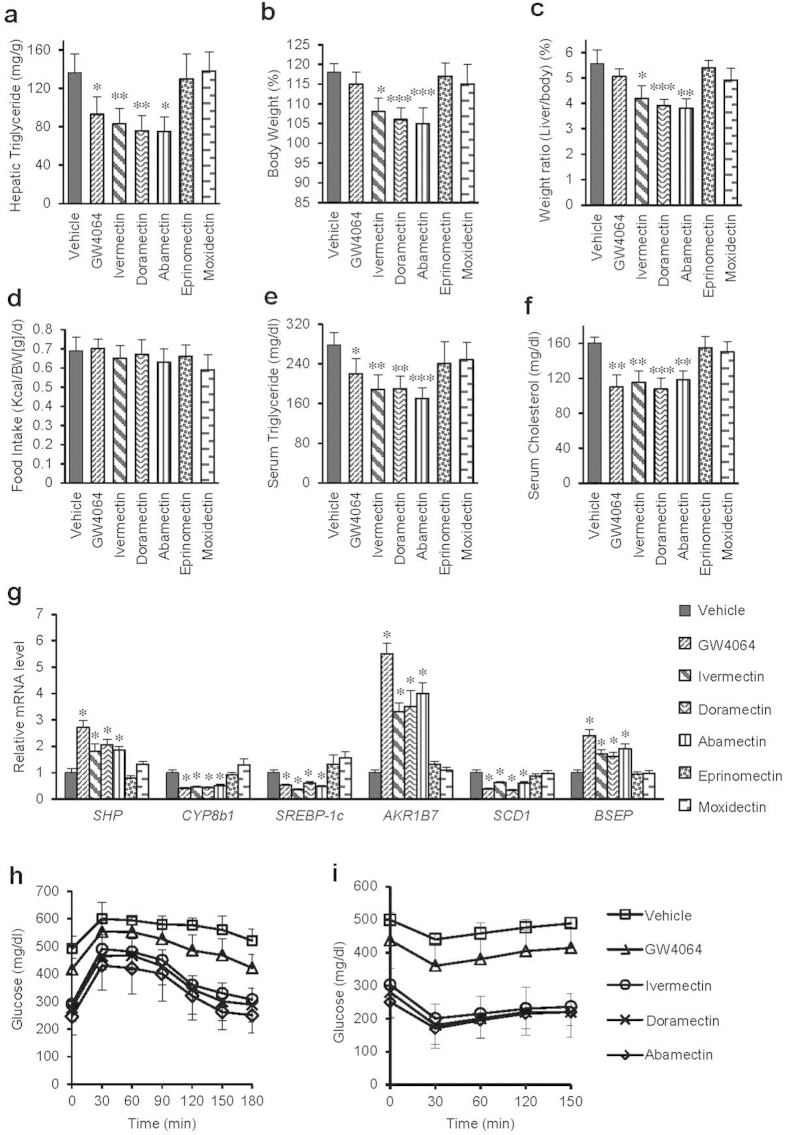
Effects of avermectin analogues on various mice metabolic parameters. (**a**) Hepatic triglyceride. (**b**) Body weight. (**c**) Liver/body weight ratio. (**d**) Food intake. (**e**) Serum triglyceride. (**f**) Serum cholesterol. (**g**) Relative mRNA levels of genes related with lipid metabolism by real-time PCR. (**h**) GTT. (**i)** ITT. n = 6 per group, Values are the means ± SEM. *p < 0.05, **p < 0.01, and ***p < 0.001 versus vehicle.

The triglyceride levels in liver tissues detected by chemical kit assay also displayed the same results with those liver sections (Fig. 3a). Notably, the body weight and liver/body weight ratio of mice treated with ivermectin, doramectin and abamectin were also significantly lower than those of mice treated with vehicle, eprinomectin and moxidectin (Fig. 3b,c), despite that all compounds had no effect on food intake (Fig. 3d). Since hypertriglyceridemia and hypercholesterolemia are closely related with liver steatosis and atherosclerosis^29^, we also tested the serum triglyceride and cholesterol levels of mice treated with compounds. The results showed that treatment with ivermectin, doramectin and abamectin significantly decreased the serum triglyceride and cholesterol levels, but treatment with eprinomectin and moxidectin did not (Fig. 3e,f). Obesity can induce elevation of blood glucose, leading to type II diabetes with insulin resistance that may also result in NAFLD by inducing hepatic fat accumulation^30^,^31^. Impressively, doramectin and abamectin treatment significantly improved glucose tolerance and insulin sensitivity of KK-Ay mice (Fig. 3h,i), which is similar to ivermectin as we reported previously^28^.

Triglyceride accumulation is due to the imbalance between triglyceride synthesis and clearance. One key gene controlling hepatic lipogenesis is sterol regulatory element-binding protein-1c (*SREBP-1c*), whose up-regulation has been implicated in occurrence of hepatic steatosis^32^,^33^, possibly by further enhancing the expression of downstream lipogenesis genes like stearoyl-CoA desaturase-1 (*SCD1*)^34^. Our quantitative PCR data revealed that ivermectin, doramectin or abamectin treatment substantially decreased the hepatic mRNA levels of *SREBP-1c* and *SCD1*, and also dramatically induced the mRNA levels of a direct target gene of FXR, *AKR1B7*, whose overexpression can lower hepatic triglyceride level (Fig. 3g). They also induced expression of small heterodimer partner (*SHP*) and bile salt export pump (*BSEP*), while decreased the levels of cytochrome P450, family 8, subfamily B, polypeptide 1 (*CYP8b1*), other three FXR target genes. However, eprinomectin and moxidectin treatment did not display these regulations. In addition, the activation of FXR by avermectins ligands also induced the expression of forkhead box m1b (*FOXM1b*) (Supplementary Fig. S1), a transcription factor related to liver cell proliferation^35^. Interestingly, enteric fibroblast growth factor 15 (*FGF15*) that is shown to benefit hepatic steatosis^36^ was also induced by the avermectins ligands of FXR in the intestine (Supplementary Fig. S2). The gene expression pattern in KK-Ay mice thus further explained the underlying molecular mechanism for the reduction of lipid accumulation by the different macrocyclic lactone analogues (Fig. 3g).

In summary, among avermectin analogues tested, ivermectin, doramectin and abamectin, but not eprinomectin and moxidectin, can effectively reduce the hepatic lipid accumulation in mice, suggesting distinct properties exist in avermectin analogues, causing their disparate potency in regulating metabolism.

### The potency of avermectin analogues in regulating metabolism correlates with their FXR binding capabilities

**Figure 4:**
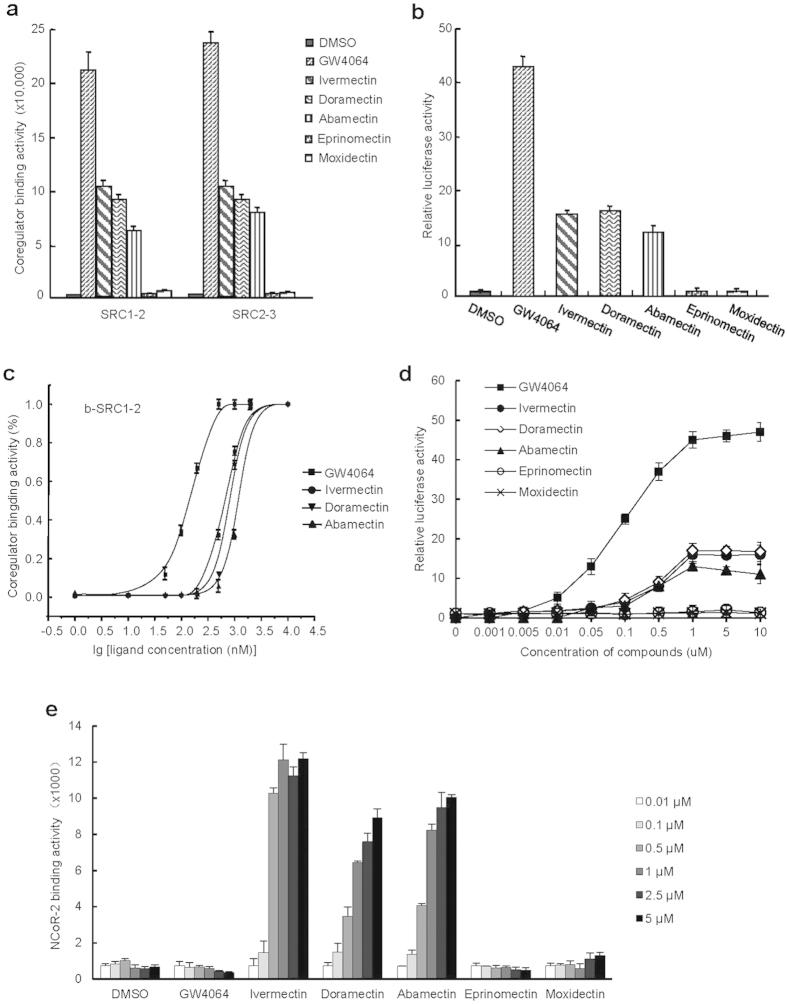
The potency of avermectin analogues in regulating metabolism correlates with their FXR binding capabilities. (**a**) Various coactivator motifs bind to FXR in response to 0.5 μM avermectin analogues or GW4064 by AlphaScreen assay. (**b**) 1 μM compounds induce FXR activity in reporter assays. (**c**) Dose responses of compounds in inducing FXR to recruit SRC1-2 coregulator binding motif by AlphaScreen assay. (**d**) Dose response of compounds in inducing the activity of FXR in reporter assays. (**e**) The recruitment of NCoR-2 to FXR in response to avermectin analogues by AlphaScreen assay. Values are the means ± SEM of three independent experiments.

To investigate the molecular mechanism of various avermectin analogues in differential regulating metabolism in mice, we tested the binding affinity of these analogues to FXR. Based on the characteristic that ligands induce nuclear receptor to recruit cofactors^37^, we used his-tagged FXR ligand binding domain (LBD) expressed and purified from BL21 (DE3) and synthetic biotin-labelled coactivator peptides including steroid receptor coactivator (SRC) 1–2 and SRC2–3, to perform the AlphaScreen assay. Interestingly, the data showed that ivermectin, doramectin and abamectin, analogues that reduced lipid accumulation in mice livers, strongly promoted the coactivators recruitment by FXR (Fig. 4a). In contrast, eprinomectin and moxidectin, analogues that did not reduce lipid accumulation in mice livers, failed to induce the recruitment of either biotin-SRC1–2 or biotin-SRC2–3 to FXR LBD (Fig. 4a). Since ligands can regulate the transcriptional activity of nuclear receptors by binding into their ligand binding pocket, we performed cell-based reporter assay to validate the results from AlphaScreen. Eprinomectin and moxidectin failed to activate the transcriptional activity of FXR (Fig. 4b). Similar to ivermectin^28^, doramectin and abamectin selectively activated the transcriptional activity of FXR using an EcRE reporter, with weaker activity than GW4064, but had no substantial impact on other nuclear receptors tested (Supplementary Fig. S3). It’s reported that LXRα antagonist can attenuate high-fat diet-induced nonalcoholic fatty liver^38^. To test whether avermectins regulate metabolism by targeting LXRs as antagonists, we performed a competitive reporter assay. The results showed that avermectins had no significant effects on the transcriptional activities of LXRs stimulated by T0901317 (Supplementary Fig. S4), demonstrating that avermectins are not LXRs antagonists.

Meanwhile, the binding affinities of these analogues to FXR were also detected by AlphaScreen and reporter assays in a dose curve manner (Fig. 4c,d). In the AlphaScreen assay (Fig. 4c), the EC50 of doramectin and abamectin were 0.5 μM and 1.25 μM, respectively, similar with ivermectin (0.4 μM), but less than that of GW4064 (0.14 μM). In the reporter assay (Fig. 4d), the EC50 of doramectin, abamectin and ivermectin were all about 0.5 μM, much less than that of GW4064 (90 nM). These results indicated that doramectin and abamectin, but not moxidectin and eprinomectin, are partial agonists for FXR, correlating very well with the functions of these compounds in reducing hepatic fat accumulation in mice.

The physiological and pharmacological actions of nuclear receptor ligands are modulated by differential recruitment of nuclear receptor coregulators, which in turn lead to the regulation of downstream nuclear receptor target genes^39^. Notably, doramectin or abamectin induced the recruitment of nuclear receptor corepressor (NCoR)-2 by FXR (Fig. 4e), which is distinct from GW4064 and similar to ivermectin^28^, another evidence for them characterized as partial agonists, in addition to their weak transcriptional activity.

**Figure 5:**
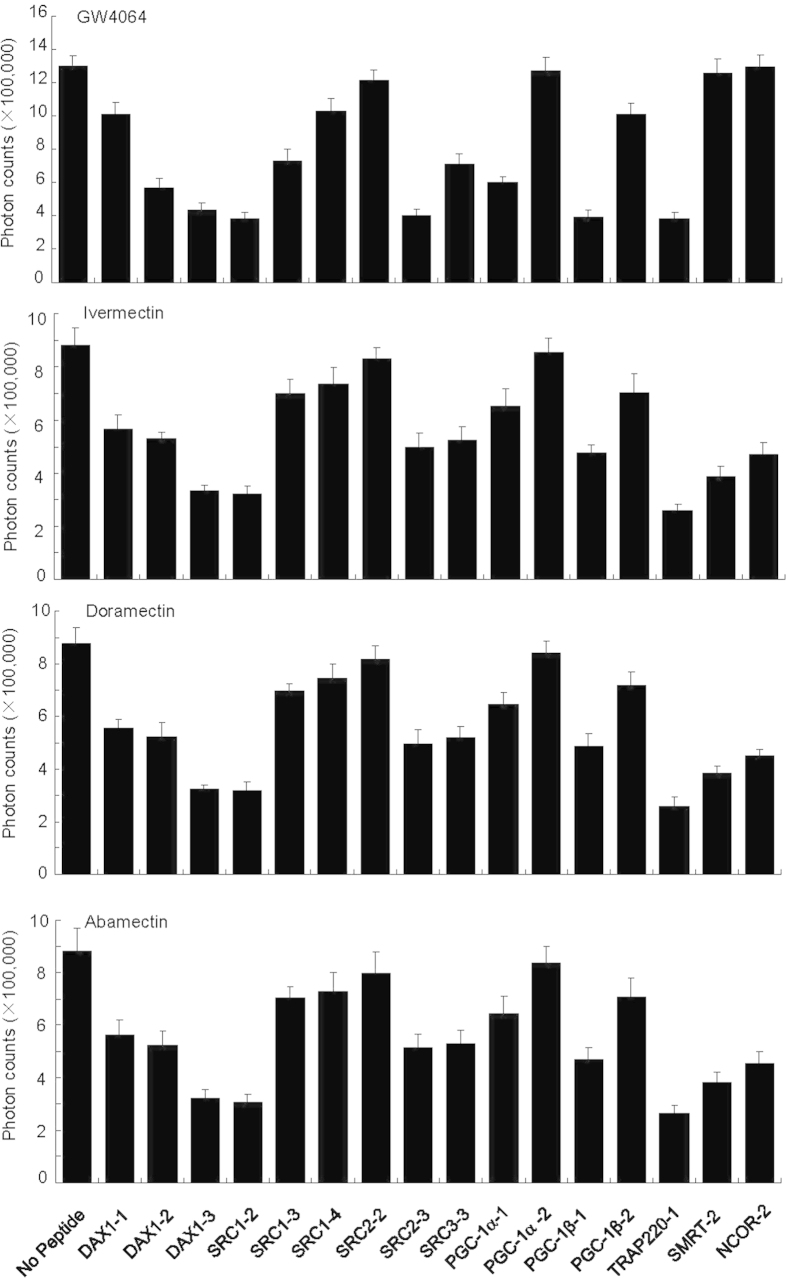
Relative binding affinity of various peptide motifs to the FXR LBD in the presence of avermectin analogues as determined by peptide competition assays. Various unlabeled peptides (20 μM) are used to compete off the binding of the biotin-labeled SRC2-3 LXXLL motif to FXR LBD in response to 1 μM ivermectin, abamectin, doramectin, or 0.5 μM GW4064, respectively. Values are the means ± SEM of three independent experiments. Sequences of peptides used in the AlphaScreen assays are as described previously^28^.

Peptide profiling is a powerful tool to detect ligand-dependent conformational properties of nuclear receptor LBDs and the correlations receptor binding with functions of nuclear receptor ligands^40^,^41^. For example, peptide profiling is particularly useful to discern the conformational differences of estrogen receptor (ER) in response to binding of agonists, antagonists and SERMs (selective ER modulators)^41^. To determine whether there is a conformational difference of FXR with various avermectin analogues, we performed peptide-profiling experiments using a panel of 16 unlabelled peptides selected from both coactivators and corepressors to compete off the binding of the third LXXLL motif of SRC2 (SRC2–3) to FXR in response to the binding of avermectin analogues (Fig. 5). The result revealed that the doramectin or abamectin bound FXR has identical peptide profiles as the ivermectin (Fig. 5), suggesting that FXR bound with these three avermectin analogues essentially adopts identical conformations. Consistent with the results above, corepressor motifs, including silencing mediator of retinoid and thyroid receptors (SMRT)-2, substantially inhibited the SRC2 binding to FXR in response to avermectin analogues, but not GW4064-bound FXR. Our results suggest a partial agonist nature of avermectin analogues with unique properties in modulating coregulator recruitment.

### The therapeutic effects of avermectin analogues to reduce lipid accumulation are FXR-dependent

**Figure 6:**
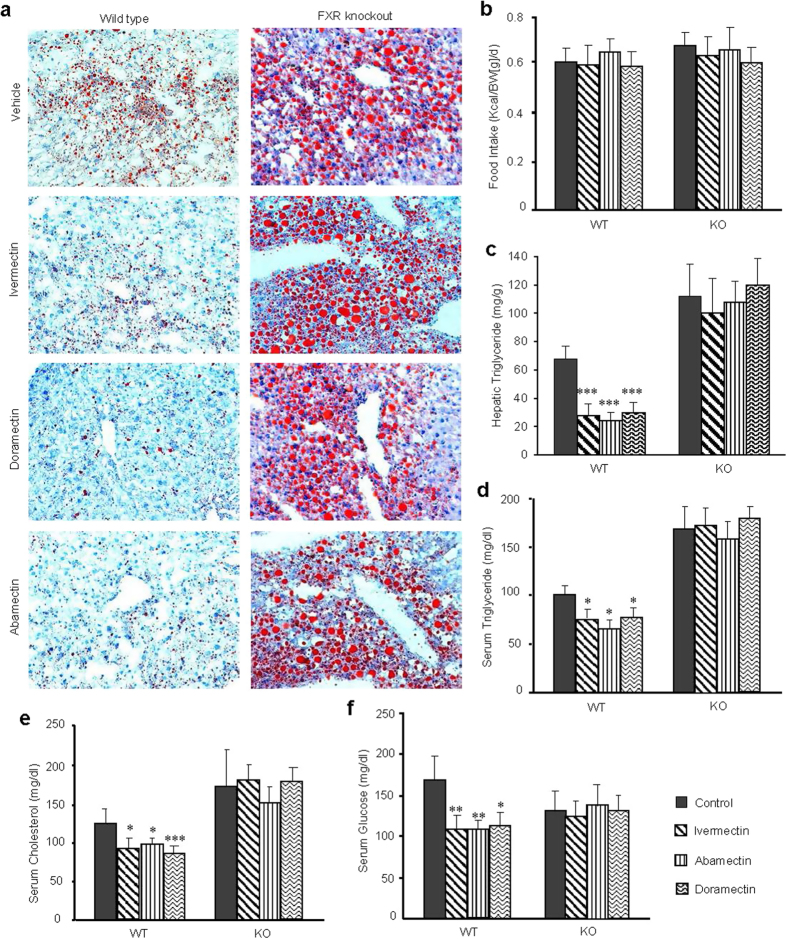
The therapeutic effects of avermectin analogues to reduce lipid accumulation were dependent on FXR. (**a**) Oil Red O staining of liver sections (original magnification, ×200). (**b**) Food intake. (**c**) Hepatic triglyceride. (**d**) Serum triglyceride. (**e**) Serum cholesterol. (**f**) Serum glucose. WT, Wild type C57BL/6J mice; KO, FXR knockout mice. n = 6 per group, Values are the means ± SEM. *p < 0.05, and ***p < 0.001 versus vehicle.

To reaffirm that the function of avermectin analogues on lipid metabolism is by directly targeting FXR signaling, we employed wild type mice and FXR gene knockout mice, both fed with high-fat diet, to study the FXR dependency of physiological effects of these avermectin analogues. As shown in Fig. 6a, the liver sections of vehicle control FXR null mice showed much larger lipid droplets and more lipid accumulation than those of the vehicle control wild type mice, which was consistent with previous reports^14^. Compared with the vehicle control wild type mice, treatment with ivermectin, abamectin or doramectin significantly reduced the amount of lipid without affecting food intake (Fig. 6a,b). In contrast, there were nearly no differences observed in FXR null mice treated with each of these compounds, demonstrating that FXR was necessary for these compounds to reduce lipid accumulation in mice liver. The data from the chemical kit assay further proved that these compounds exactly significantly decreased the triglyceride levels in wild type mice, but not in FXR null mice even though the triglyceride levels were much higher than that in the wild type mice (Fig. 6c). In addition, treatment with all three compounds also decreased the serum triglyceride levels of mice in a FXR dependent manner (Fig. 6d). Similar to ivermectin, doramectin or abamectin treatment also lowered the serum cholesterol and glucose levels in a FXR dependent manner (Fig. 6e,f). The expression pattern of FXR target genes *SHP*, *BSEP*, solute carrier family 51 beta subunit (*OSTβ*) and *AKR1B7* in primary hepatocytes from wild type mice and FXR gene knockout mice further confirmed the FXR-dependent manner of avermectin analogues (Supplementary Fig. S5). These results demonstrated that the therapeutic effects of avermectin analogues to regulate metabolism, including lipid, cholesterol and glucose metabolism, are dependent on nuclear receptor FXR.

### The structural basis for the selective binding of avermectin analogues with FXR

**Figure 7:**
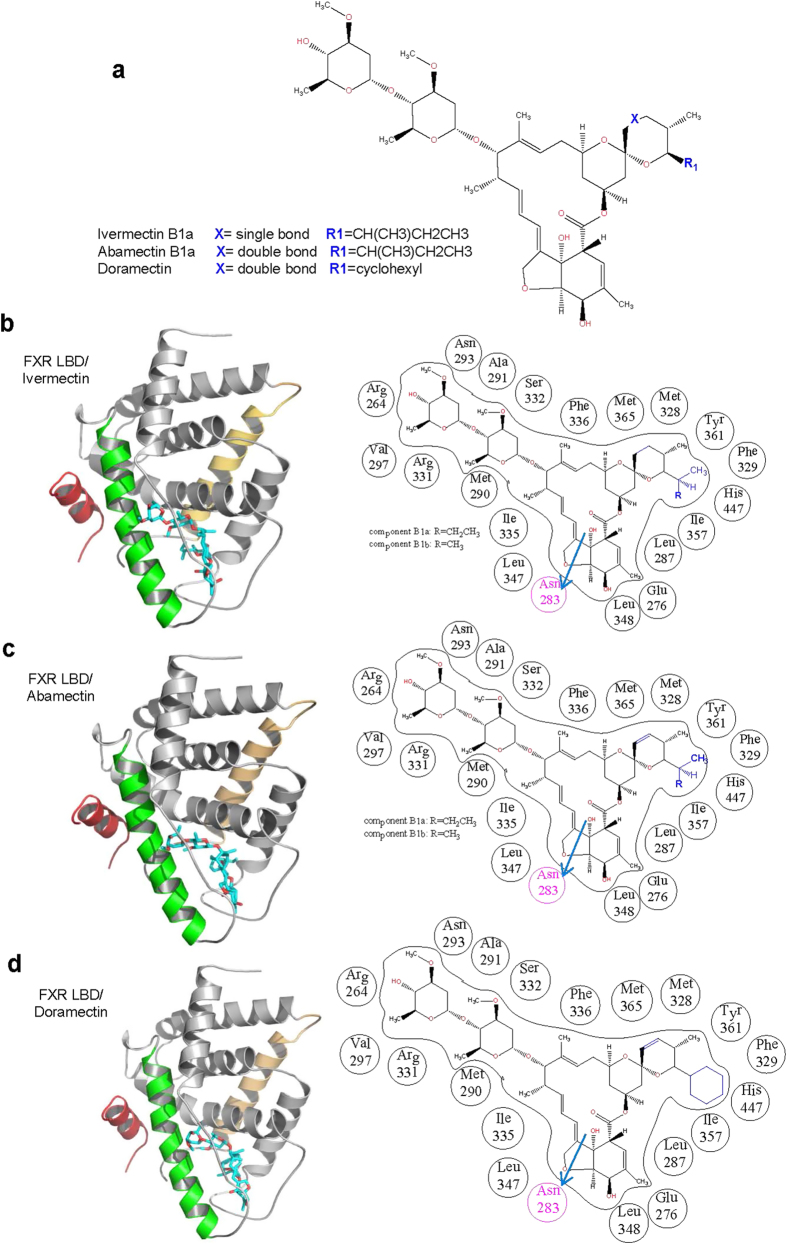
Protetin-ligand docking of avermectin analogues in the ligand binding pocket of FXR. (**a**) A general chemical structure of ivermectin, abamectin and doramectin. (**b–d**) Left:The structure of FXR bound with ivermectin and the docking structures of the FXR docked with abamectin and doramectin in ribbon representation. Right: Schematic representation of interactions between FXR and avermectin analogues. Hydrogen bonds are indicated by arrows from proton donors to acceptors. The helix 3 of the FXR LBD is highlighted in green, the NCoR-2 motif is in brownish red.

As shown in Fig. 7a, the only difference of the molecular structure between abamectin and ivermectin is the double bond and single bond at C22-C23. Doramectin differs to abamectin in the R1 group with a cyclohexane group. Eprinomectin differs to abamectin with an acetamide instead of hydroxyl group at C4” site. According to our previously published X-ray crystal structure (PDB ID: 4WVD)^28^, ivermectin anchors in the ligand binding domain (LBD) of FXR mainly by hydrophobic interactions with the lipophilic amino acid residues and two hydrogen bond interactions with the carbonyl oxygen on the main-chain of Arg264 and the amide carbonyl oxygen on the side-chain of Asn283, respectively (Fig. 7b). From the structural docking models in Fig. 7c, the double bond of abamectin at C22-C23 does not affect its binding to FXR compared with ivermectin binding. The cyclohexane group of doramectin in R1 group is in the spacious center of the ligand binding pocket, and it’s also hydrophobic similar to ivermectin, which does not affect its binding to FXR too (Fig. 7d). For eprinomectin, despite that the main part of eprinomectin matches well in the ligand binding pocket, the displaced acetamide group has several adverse effects on its binding in the hydrophobic ligand binding pocket. It increases the polarity of the ligand and the acetamide group of eprinomectin interferes with the side chain of Asn293 and Arg264, or even the whole helix3 and the loop between helix1 and helix2, leading to the disruption of the hydrogen bond with the amide carbonyl oxygen on the main-chain of Arg264. As for moxidectin, the loss of the bulky heterocycle group results in the insufficient hydrophobic interactions, which makes it difficult to fix well in the hydrophobic pocket of FXR.

**Figure 8:**
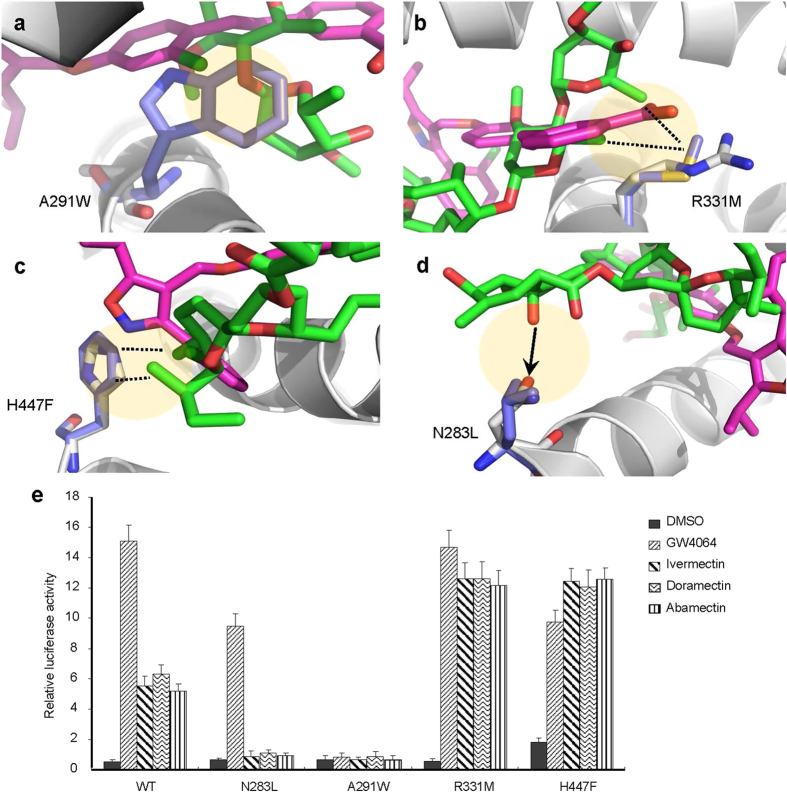
Functional correlation of the avermectins/FXR interactions. (**a–d**) Molecular determinants of the interaction between FXR with avermectins. Overlays of avermectin (green) and GW4064 (salmon red) in the FXR structure (grey). The selected residues important for ligand interaction are shown in stick representation with wild-type and mutant depicted in white and blue, respectively. The hydrogen bonds are shown with arrows. The potential hydrophobic interactions, if the corresponding mutations are made as indicated in (e), are shown in dashed lines. (**e**) Differential effects of mutations of key FXR residues on its transcriptional activity in response to 1 μM avermectin analogues in reporter assays. Values are the means ± SEM of three independent experiments.

To validate the roles of pocket residues in macrocyclic lactones binding and FXR activation, we mutated several key FXR residues that contact different groups of doramectin and abamectin and tested the transcriptional activity of these mutated FXR in response to the compounds in cell-based reporter assays. As shown in Fig. 8a, A291W mutation reduced the size of FXR pocket for the long heterocycle group of avermectins, thereby preventing the binding of these bulky avermectin ligands. As expected, this mutation abolished the transcriptional activity of FXR by doramectin, abamectin, ivermectin, and also GW4064 (Fig. 8e). R331M mutation was supposed to strengthen the hydrophobic interactions by displacing the corresponding residue of polar arginine to a hydrophobic methionine (Fig. 8b). Accordingly, this mutation increased the ability to be activated by avermectin ligands (Fig. 8e). For H447F mutation, the corresponding residue of histidine with a side chain of imidazole group was replaced by the nonpolar phenylalanine with a side chain of benzene ring (Fig. 8c). In the same way, this mutation increased the FXR activity response to doramectin and abamectin due to the consolidation of hydrophobic interactions (Fig. 8e). N283L mutation disrupts a key hydrogen bond between FXR and avermectin compounds (Fig. 8d). Accordingly, this mutant abolished avermectins-mediated FXR transcriptional activity but retained the ability to be activated by GW4064 (Fig. 8e). Taken together, our mutagenic analysis showed distinct results on the activation of FXR by different avermectins and GW4064, highlighting the differential roles of FXR pocket residues in recognizing various ligands.

## Discussion

Avermectins are main antiparasitic drugs in the global market. Due to the excellent efficacy and the huge market needs, a series of analogues have been semi-synthesized based on avermectin^25^. The milbemycins, chemically related to the avermectins, are a group of macrolides with a longer half-life than avermectins^27^. These antiparasitic drugs share similar mechanisms of action by blocking the nerve systems of parasites through binding the gamma-aminobutyric acid (GABA) receptor, leading to parasites death ultimately^26^,^42^,^43^. Unlike distributing at the neuromuscular junctions and the central ventral cords in nematodes, GABA receptors are found primarily in the brain of mammals. Avermectins and milbemycins are not able to cross the blood-brain barrier in mammals at therapeutic doses, which ensures the safety of their use in mammals^26^,^43^.

In our last study, we uncovered a specific receptor for ivermectin with high affinity and selectivity in mammalians^28^. By binding with the nuclear receptor FXR, ivermectin treatment decreased the serum glucose and cholesterol levels in diabetic mice. Since the avermectin analogues have a close structural relationship to ivermectin, this finding raises the question whether all the avermectin analogues can regulate metabolism. In view of the crucial roles of FXR in lipid metabolism, we focused on the study of therapeutic effects of ivermectin on NAFLD in this study. To explore mechanisms of ivermectin action and more options for pharmacological intervention, we furthered our investigations on avermectin analogues by selecting abamectin, doramectin and eprinomectin from avermectins, and moxidectin from milbemycins.

Our study indicates that ivermectin possesses remarkable beneficial effects on the hepatic lipid accumulation, but not all the selected macrocyclic lactones had similar effects as ivermectin. Among the selected analogues, eprinomectin and moxidectin treatment did not show any therapeutic effects on high-fat diet fed KK-Ay mice with obesity and diabetes. Doramectin and abamectin exhibited excellent effects similar to ivermectin on the typical symptoms of NAFLD. They dramatically reduced the lipid accumulation in the livers of the obese diabetic mice, decreased the liver/body weight ratio together with the loss of body weight. They also lowered the serum triglyceride and cholesterol levels, as well as the serum glucose levels and improved insulin sensitivity. More importantly, these functions of the three avermectins were dependent on the nuclear receptor FXR, as they failed any improvement effects on the mentioned symptoms in mice with FXR deficiency. Mechanistic studies have provided a clear clue for the disparate functions among different avermectin analogues. Doramectin and abamectin, the same as ivermectin, induced FXR to recruit cofactors, and activated the FXR transcriptional activity. In contrast, eprinomectin and moxidectin did not induce recruitment of any cofactors including coactivators and corepressors to FXR, or affect the transcriptional activity of FXR. Therefore, avermectin analogues that bind FXR may be used to treat NAFLD.

We further revealed doramectin, abamectin and ivermectin as FXR partial agonists, which may provide a molecular mechanism for the unique properties of these antiparasitic FXR ligands. First, the ability for the transcriptional activity of FXR by these compounds was weaker than the full agonist, GW4064. The second reason was the obviously lower binding affinities to FXR, evidenced by their higher EC50 in transactivating FXR. Most importantly, they can induce the recruitment of corepressor NCoR-2, which is distinct from full agonists, like GW4064. Partial agonists have also been reported to promote recruitment of corepressors in other nuclear receptors^44^,^45^. For example, vitamin D receptor (VDR) ligands can promote the recruitment of both coactivator SRC1 and corepressors SMRT and NCoR^44^. Both NCoR and SMRT were observed in association with agonist and antagonist complexes of glucocorticoid receptors^45^. Accordingly, the FXR ligands avermectins in this study were characterized as partial agonists, different from classical full agonist or antagonist. Our study further suggested that the transcriptional activity of nuclear receptors does not necessarily correlate with the metabolism regulation^46^.

Aside from diet and physical exercise as a basal universal approach, no specific drug is actually available to treat liver NAFLD. As such, specific liver drugs for NAFLD are pressingly needed along with the worldwide epidemic of obesity and their strong association with metabolic syndrome and cancer. Given the importance of FXR in liver, it’s hopeful to find some potential ligands for treating NAFLD. Although GW4064 is a useful tool compound for investigating the functions of FXR, some flaws including solubility, potentially toxic stilbene pharmacophore, UV light instability and poor pharmacokinetics (t_1/2_ < 1 h in rat) limited its use as a candidate of drug^47^,^48^,^49^. Avermectins, however, displayed their advantages in these aspects. The safety of the avermectin analogues has been verified because of their decades of use to treat parasitic infections in animals and human. The elimination half-lives of avermectins *in vivo* are about 18 h, even up to 8 days in some formulations^50^,^51^, which might also explain the striking effects of avermectins in a low dose compared with that of GW4064 in a high dose. Secondly, the effects of avermectins on metabolism may be due to tissue-selective targeting of FXR in intestine besides in liver, because avermectins showed similar or stronger regulation of FXR target genes for avermectins than GW4064 in intestine (supplementary Fig. S2), compared to those in liver (Fig. 3g). Thirdly, the unique ability of avermectins in inducing the recruitment of corepressors to FXR may also contribute to their therapeutic effects, which remains to be further studied. Given the excellent therapeutic effects on metabolism, the FXR-ligand avermectins can provide a novel class of promising drugs to treat metabolic syndromes including NAFLD, NASH, hyperlipidemia, and diabetes. Additionally, the structural differences between FXR-ligand avermectin analogues and the non-FXR-ligand avermectin analogues provide new insight and strategies to design drugs targeting nuclear receptor FXR.

## Methods

### Animals, diets, and compounds

10 weeks age KK-Ay mice and C57BL/6J, homozygous FXR deficient (FXR^–/–^) mice were maintained under environmentally controlled conditions with free access to standard chow diet and water as described previously^28^. Animal experiments were conducted in the barrier facility of the Laboratory Animal Center, Xiamen University, approved by the Institutional Animal Use and Care Committee of Xiamen University, China, and the methods were carried out in accordance with the approved guidelines. Mice were fed with high fat diet containing 60% fat, 20% protein and 20% carbohydrate (Research Diets, D12492, New Brunswick, USA), and the food intake were measured every second day. Mice were i.p. injected with either vehicle (40% of 2-hydroxypropyl-β-cyclodextrin (HBC), Sigma, USA) or vehicle containing GW4064 (30 mg/kg, Sigma) or avermectin analogues (1.3 mg/kg) (International Laboratory, USA) once a day for 14 days. After 6 h fasting, mice were weighed and sacrificed. Livers were weighed and taken photos, and part of each liver was fixed in 4% paraformaldehyde. Liver histology characterization was analyzed by haematoxylin and eosin (H&E) staining with paraffin-embedded sections by standard procedures. Other tissues were collected and frozen in liquid nitrogen for use, and serums were collected to measure metabolite parameters.

### Oil red O staining

Fresh liver tissues were embedded in optimum cutting temperature compound (OCT) and cryosectioned. The sections were fixed in 4% paraformaldehyde in PBS, and were stained with 0.3% oil red O according to standard procedures.

### GTT and ITT

Mice treated with compounds for 10 days were fasted for 6 h with free access to water. For the glucose tolerance test (GTT), 1 g/kg of glucose was i.p. injected and blood glucose was measured with the Accu-Check Performa (Roche Applied Science, Mannheim, Germany) at 0, 30, 60, 90, 120,150 and 180 min. For the insulin tolerance test (ITT), 1 U/kg of recombinant human insulin (Novolin 30R; Novo Nordisk, Bagsvaerd, Denmark) was i.p. injected, and blood glucose was measured at 0, 30, 60, 120 and 150 min after insulin injection.

### Quantification of serum glucose, triglyceride and cholesterol levels

Serum glucose was analyzed using glucose oxidase method (Applygen, Beijing, China). Serum cholesterol and triglyceride were analyzed using Cholesterol Assay Kit and Triglyceride Assay Kit (Bioassay Systems, USA), respectively. Liver triglyceride was analyzed using Tissue triglyceride assay kit (Applygen, Beijing, China).

### Real-Time PCR

Total RNA was isolated using Tissue RNA kit (Omega Bio-Tek, GA). The first strand cDNA were obtained by TAKARA reverse transcription kit. Real-time quantitative PCR were performed on a CFX96™ Real-Time PCR Detection System (Bio-Rad) using SYBR Premix Ex TaqTM (TAKARA). Relative mRNA expression levels were normalized to actin levels.

### Cofactor binding assays

The binding of the cofactor peptide motifs to FXR LBD in response to ligands was determined by AlphaScreen assays using a hexahistidine detection kit from Perkins-Elmer (USA) as described before^28^. The FXR LBD protein was purified as described previously^28^. The peptides with an N-terminal biotinylation are listed below: SRC1–2, SPSSHSSLTERHKILHRLLQEGSP; SRC2–3, QEPVSPKKKENALLRYLLDKDDTKD; NCoR-2, GHSFADPASNLGLEDIIRKALMGSF.

### Transient transfection assay

COS-7 cells were maintained in DMEM containing 10% fetal bovine serum and were transiently transfected using Lipofectamine 2000 (Invitrogen, USA) as described previously^28^. The mutant FXR plasmids were created using the Quick-Change site-directed mutagenesis kit (Stratagene, USA). The reporter assay was performed as described previously^28^.

### Protein-ligand docking

The protein-ligand docking was based on the crystal structure of FXR bound with ivermectin(PDB ID: 4WVD)^28^. One monomer of the dimer crystal structure was isolated and the crystallized ligand was removed. The protein-ligand docking was performed using AutoDockVina software^52^ with standard parameters.All figures were rendered using PyMOL.

### Statistical analysis

Values were expressed as mean ± standard error of mean (SEM). Statistical differences were calculated by Student’s *t* test. Statistical significance was shown as *p < 0.05, **p < 0.01 or ***p < 0.001.

## Additional Information

**How to cite this article**: Jin, L. *et al*. Selective targeting of nuclear receptor FXR by avermectin analogues with therapeutic effects on nonalcoholic fatty liver disease. *Sci. Rep*. **5**, 17288; doi: 10.1038/srep17288 (2015).

## Supplementary Material

Supplementary Information
