# Supplementary material for: Selective targeting of nuclear receptor FXR by avermectin analogues with therapeutic effects on nonalcoholic fatty liver disease
Source: Sci Rep. 2015 Dec 1;5:17288. doi: 10.1038/srep17288 (PMC4664883; doi:10.1038/srep17288)
Supplement: Supplementary Information [file srep17288-s1.pdf]

Supplementary Information

Selective targeting of nuclear receptor FXR by avermectin analogues with therapeutic effects on nonalcoholic fatty liver disease

Lihua Jin[‡]*, Rui Wang[†], Yanlin Zhu[†], Weili Zheng[†], Yaping Han, Fusheng Guo, Frank Bin Ye, and Yong Li*

State Key Laboratory of Cellular Stress Biology, Innovation Center for Cell Signaling Network, School of Life Sciences, Xiamen University, Fujian 361005, China

[†]These authors contributed equally to this work
*Correspondence author. Yong Li (yongli@xmu.edu.cn) or Lihua Jin (jinlh@xmu.edu.cn)

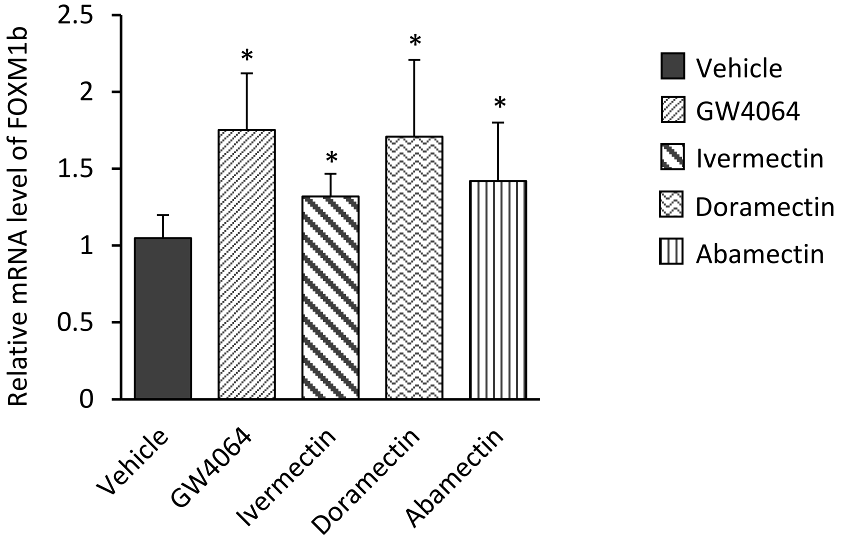

Supplementary Fig. S1. Relative mRNA levels of *FOXM1b* in the liver tissues of KK-Ay mice treated with different compounds. N=6 per group, Values are the means ± SEM. *p<0.05 versus vehicle.

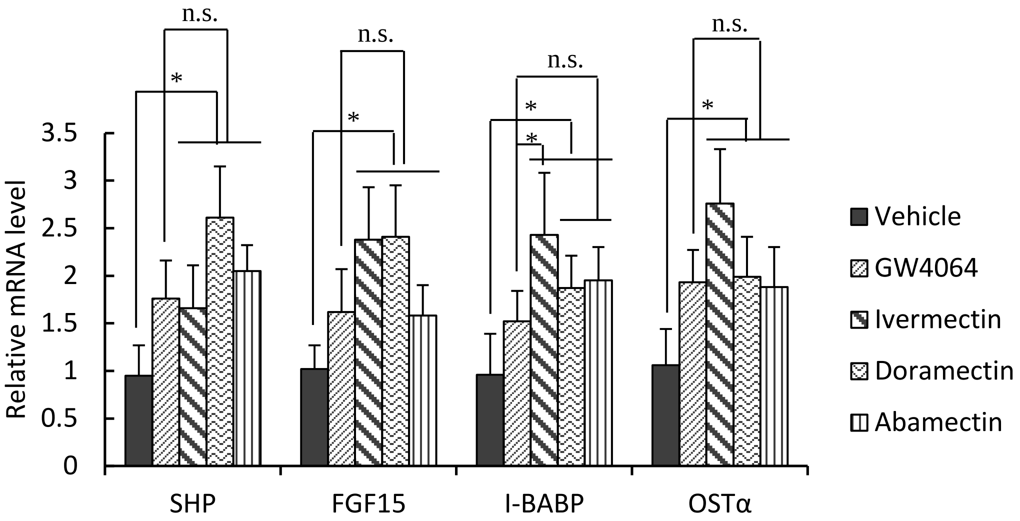

Supplementary Fig. S2. Relative mRNA levels of FXR target genes in the intestine tissues of KK-Ay mice treated with different compounds. N=6 per group, Values are the means ± SEM. *p<0.05 versus vehicle control. n.s. means no significant difference.

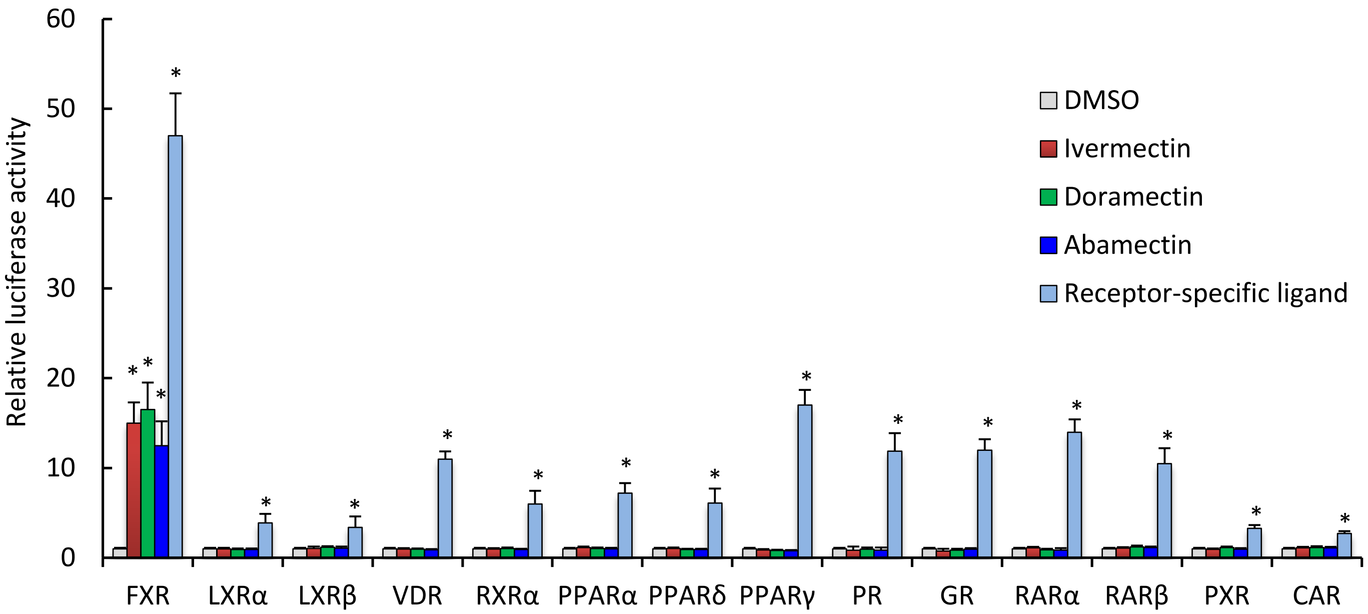

Supplementary Fig. S3. Receptor-specific transactivation by avermectins.
COS-7 cells were cotransfected with plasmids encoding full-length nuclear receptors and their cognate response reporters as follows: human FXR with EcRE-Luc, human LXR(α and β) with LXRE-luc; human VDR with VDRE-luc; Human RXRα with RXRE-luc; human PPARs (α, δ, and γ) with PPRE-Luc; human GR or PR with the MMTV-Luc; human RARα or RARβ with βRE-Luc; human PXR and CAR with PBRE-luc. 5 h after transfection, cells were treated with DMSO, 10 μM avermectins or ligands specific for each receptor: FXR, 0.5 μM GW4064; LXRα, 5 μM T0901317; LXRβ, 5 μM T0901317; VDR, 0.1μM 1α, 25-dihydroxyvitamin D3; RXRα, 1 μM all-trans-retinoic acid; PPARα, 1 μM GW590735; PPARδ, 1 μM GW0472; PPARγ, 1 μM rosiglitazone; GR, 0.1 μM dexamethasone; PR, 0.1 μM progesterone; RARα and RARβ, 1 μM all-trans-retinoic acid; PXR, 10 μM rifampicin; CAR, 5 μM CITCO. Cells were harvested 24 h later for the luciferase assays. Luciferase activities were normalized to renilla activity co-transfected as an internal control. Values are the means ± SEM of three independent experiments. *p<0.001 versus DMSO control.

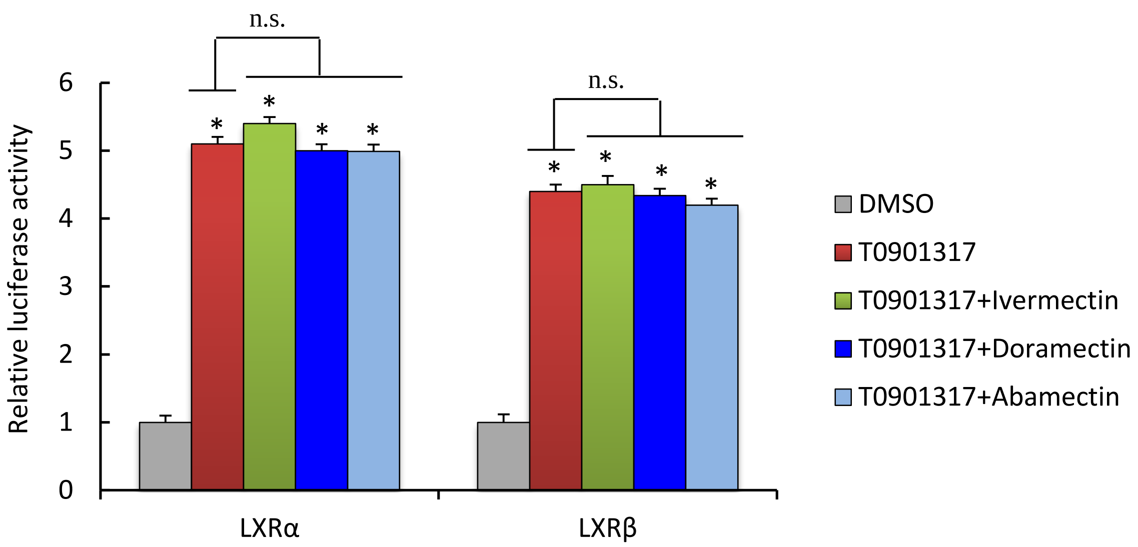

Supplementary Fig. S4. Avermectins have no effects on the transcriptional activities of LXRs stimulated by T0901317.

COS-7 cells were cotransfected with pG5-Luc reporter together with pBind-LXRα LBD or pBind-LXRβ LBD that express LXRs LBD protein fused with the Gal4 DNA-binding domain. 5 h after transfection, cells were treated with DMSO, 5 μM T0901317 or 5 μM T0901317 combined with 10 μM avermectins. Cells were harvested 24 h later for the luciferase assays. Luciferase activities were normalized to renilla activity co-transfected as an internal control. Values are the means ± SEM of three independent experiments. *$p<0.001$ versus DMSO control, n.s. means no significant difference.

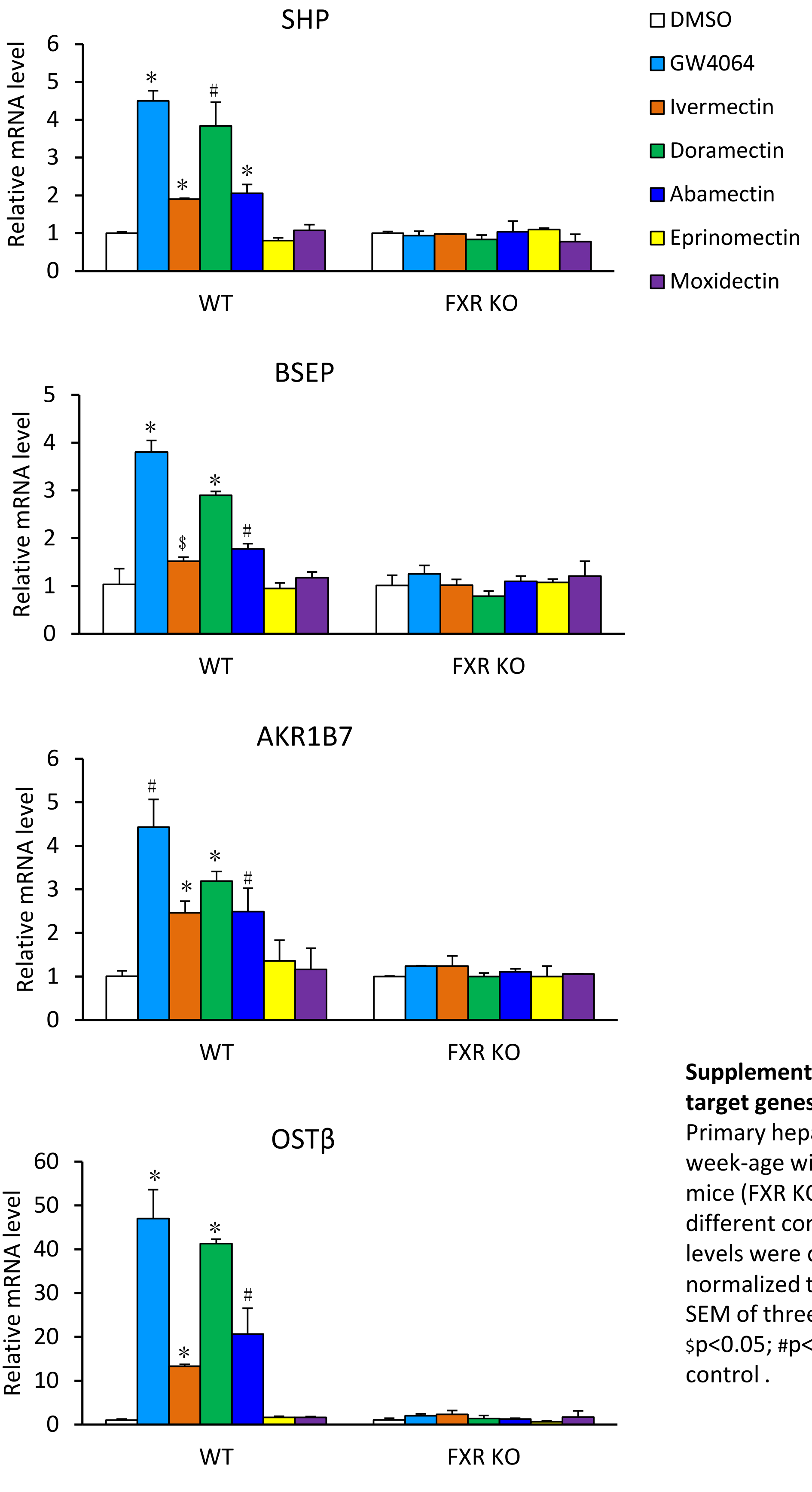

Supplementary Fig. S5. Expression of FXR target genes in primary hepatocytes. Primary hepatocytes were extracted from 8-week-age wild type mice (WT) or FXR knockout mice (FXR KO), and treated with 1 μM of different compounds for 24 h. Relative mRNA levels were quantified by real-time PCR and normalized to actin. Values are the means ± SEM of three independent experiments. $p<0.05; #p<0.01; *p<0.001, versus DMSO control .
